# Editorial: Emotion processing in autism spectrum disorders

**DOI:** 10.3389/fpsyg.2023.1141824

**Published:** 2023-02-21

**Authors:** Denise Davidson, Nicole Russo-Ponsaran, Maaike Marijn van Rest, Angela Scarpa

**Affiliations:** ^1^Department of Psychology, Loyola University Chicago, Chicago, IL, United States; ^2^Department of Psychiatry and Behavioral Sciences, Rush Medical College, Rush University, Chicago, IL, United States; ^3^Department of Clinical Child and Family Studies, Vrije Universiteit Amsterdam, Amsterdam, Netherlands; ^4^Psychology Department, Virginia Tech University, Blacksburg, VA, United States

**Keywords:** emotion processing and regulation, autism spectrum disorder, autism, emotion recognition, alexithymia, externalizing and internalizing behavior

Autism spectrum disorder (ASD) is a neurodevelopmental condition characterized by differences in social communication and restrictive and repetitive interests. Although progress has been made in understanding its phenomenology, underlying mechanisms for core and co-occurring difficulties remain elusive. Emerging evidence suggests that emotion processing and regulation play a critical role in the challenges experienced by autistic individuals. This special issue provides current thinking related to methods, research, and practice in the field of emotion processing and regulation in ASD, and to integrate study findings to elucidate underlying mechanisms. With a global perspective, article coverage includes (1) explorations of the neurological underpinnings of emotion processing differences in individuals with and without autism, (2) emotion recognition and related issues (i.e., theory of mind, temperament) in siblings of autistic children, (3) examinations of the interplay between emotion, anxiety, sensory sensitivities and internalizing and externalizing challenges, and (4) emotion processing in real-world applications, including driving and adjustment to college.

Examining neurological underpinnings, Safar et al. used magnetoencephalography to investigate shared and distinct patterns of functional connectivity to happy and angry faces in ASD or attention-deficit/hyperactivity disorder (ADHD) compared with typical development (TD). They found reduced functional connectivity in the beta band in both the ASD and ADHD groups compared to TD, with the largest reduction in the ADHD group compared to both other groups. Greater connectivity was found in the ADHD and TD groups to happy faces, while the opposite pattern was found for the ASD group. These findings suggest diverse frequency and emotion-specific patterns of functional connectivity in youth with and without neurodevelopmental disorders.

Hogan et al. examined pupillary responses as a measure of autonomic arousal in response to emotional faces in undiagnosed parents of autistic compared to non-autistic children. Pupillary responses to emotional stimuli were differentially linked to lower pragmatic language and social cognition scores in parents. Moreover, in a subset of the sample, peak pupillary responses were significantly correlated between parents and their autistic children. The findings point to pupillary response, reflecting underlying emotion processing or regulation mechanisms, as a possible genetic biomarker related to autism.

With regard to emotion recognition and related issues (e.g., anxiety), Sacrey et al. investigated relations between early temperament and later internalizing and externalizing behavior problems in younger siblings of autistic children. Multi-method assessment of temperament profiles in infancy and in toddlerhood significantly predicted internalizing and externalizing problems at age 5. Temperament was thus found to be linked to later mental health problems. These findings provide directions for prevention of mental health problems in children with increased probabilities of autism.

Uljarevic et al. compared the performance of autistic children, unaffected siblings and TD controls on affect recognition (AR) and theory of mind (ToM) tasks. Overall, autistic children showed significantly poorer AR and ToM when compared to siblings and TD children; no differences persist among groups when matched on intellectual ability (FSIQ). The authors conclude that FSIQ, rather than group membership, may have more important effects on emotion recognition and theory of mind performance. They also suggest that future work utilize more sensitive or implicit measurement techniques or broader test batteries and engage in more specific strength and weakness profiling in participants.

Normansell-Mossa et al. tested two competing theories that may explain anxiety in autistic and non-autistic adults. Their results supported an autism-first model, suggesting that sensory sensitivity and sensory seeking behaviors increase intolerance of uncertainty and resulting anxiety. These findings have implications to enhance supportive therapies for understanding and managing uncertainty and anxiety-related mental health concerns in autistic people.

In children, Tsuji et al. explored the interplay between sensory symptoms and internalizing problems (e.g., anxiety). Study findings revealed that autistic traits and sensory symptoms were distributed as a continuum in children with and without autism. Moreover, sensory symptoms in school life mediated the relation between sensory processing. Tsuji et al. suggest that developing a support system for children that specifically reduces suffering due to sensory problems would be valuable in general education settings.

In application research, Fok et al. explored emotional barriers to driving in self-identified autistic teens and young adults. Autistic compared to non-autistic participants self-reported more impulse control difficulties and heightened stress reactions, which were in turn associated with perceived driving difficulty in the entire sample. The findings suggest that over-reactivity to negative affect explains some of the barriers to driving in the autistic population, which has implications for obtaining a driver's license. Finally, Davidson and Morales examined the relations between alexithymia (difficulties in identifying emotional states), ASD symptomatology, and trait emotion intelligence (EI) in college students. Alexithymia was negatively related to trait EI—a multifaceted concept that captures emotional competencies. Additionally, both alexithymia and ASD symptomatology accounted for unique variance in trait EI. Nevertheless, only trait EI was a significant predictor of adjustment to college. The authors suggest that support programs that develop trait EI skills may improve the college experience for autistic students.

Collectively, contributing authors' use of multiple levels of analysis from neurobiological to psychosocial constructs (e.g., anxiety, temperament, emotional intelligence) to real-world applications, provides a unique lens to view the impact of emotion processing and regulation in autism. Additionally, these papers remind us of the importance of considering not only the autistic individual, but also familial connections, for better understanding underlying mechanisms and correlates. Future research can build on these findings to inform assessment of challenges faced by autistic people and to develop targeted supports promoting their mental health and wellbeing.

To conclude, we are grateful to the authors whose work provides significant additions to our knowledge about emotion processing and emotion regulation by individuals across the autism spectrum as well as their family members. We are also indebted to the *Frontiers in Psychology* staff who worked with us at all stages of the publication process and the reviewers and outside editors whose comments, suggestions and insight contributed significantly to the quality of this issue.

## Author contributions

DD organized the editorial and presentation of articles, wrote reviews of four articles appearing in the issue, wrote the opening and closing content of the article, and edited the article. AS wrote reviews of two articles and contributed to the opening and closing content. NR-P and MVR wrote reviews of articles and edited the content of the article. All authors contributed to the article and approved the submitted version.

